# Quantum AI for psychiatric diagnosis: enhancing dementia classification with quantum machine learning

**DOI:** 10.3389/fpsyt.2025.1648060

**Published:** 2025-11-26

**Authors:** Javaria Amin, Muhammad Umair Ali, Muhammad Zubair Islam, Seung Won Lee

**Affiliations:** 1 Department of Computer Science, Rawalpindi Women University, Rawalpindi, Pakistan; 2 Department of Artificial Intelligence and Robotics, Sejong University, Seoul, Republic of Korea; 3 Department of Precision Medicine, Sungkyunkwan University School of Medicine, Suwon, Republic of Korea; 4 Department of Metabiohealth, Sungkyunkwan University, Suwon, Republic of Korea; 5 Personalized Cancer Immunotherapy Research Center, Sungkyunkwan University School of Medicine, Suwon, Republic of Korea; 6 Department of Artificial Intelligence, Sungkyunkwan University, Suwon, Republic of Korea; 7 Department of Family Medicine, Kangbuk Samsung Hospital, Sungkyunkwan University School of Medicine, Seoul, Republic of Korea

**Keywords:** dementia, deep learning, quantum machine learning, features, classification

## Abstract

Early detection of dementia is a key requirement for effective patient management. Therefore, classification of dementia is pertinent and requires a highly accurate methodology. Deep learning (DL) models process immense amounts of input data, whereas quantum machine learning (QML) models use qubits and quantum operations to enhance computational speed and data storage through algorithms. QML is a research domain that investigates the interactions between quantum computing concepts and machine learning. A quantum computer reduces training time and uses qubits that play a vital role in learning complex imaging patterns, unlike convolutional kernels. The proposed study focused on imaging data and QML because they are more efficient and accurate than ML/DL for practical applications. Therefore, a hybrid quantum-classical convolutional neural network (QCNN) is proposed that integrates both quantum and classical learning paradigms. In the proposed framework, MRI images are pre-processed through resizing and normalization, followed by the extraction of a region of interest (ROI) from the center of each image. Within the ROI, a 2×2 patch is passed to a quantum circuit, where pixel values are encoded as qubits using rotation gates (RY). A parameterized quantum circuit (PQC) with entangling layers computes expectation values to generate a quantum feature map, which is then utilized as input to the classical CNN. To further improve generalization, a knowledge distillation (KD) framework is employed, where a teacher model (a deeper CNN with high representational capacity) guides a student model (the QCNN), transferring soft-label information via a temperature-scaled softmax. This setup enables the student model to learn more discriminative features while maintaining efficiency. Comprehensive experiments are conducted on benchmark ADNI-1, ADNI-2, and OASIS-2 MRI datasets, and results are reported both with and without KD. Without KD, the QCNN achieves strong performance with accuracies of 0.9523 (ADNI-1), 0.9611 (ADNI-2), and 0.9412 (OASIS-2). With KD, the student model demonstrates enhanced sensitivity to challenging classes, achieving an accuracy of up to 0.9978, surpassing state-of-the-art approaches. Combining quantum feature extraction with teacher-student knowledge transfer yields a scalable and highly accurate framework for dementia classification in clinical practice.

## 1 Introduction

The term “dementia” encompasses a wide range of symptoms related to a decline in memory and cognitive abilities. Dementia occurs when nerve cells in the brain are damaged. According to the World Health Organization (WHO) statistical report, approximately 10 million cases are reported annually (1). Depending on a person’s health and other factors, dementia has different effects on different individuals. Dementia is classified into different grades based on the signs and symptoms. In the early stages, there is an inability to track time, memory loss, and an incapacity to monitor one’s own time. The moderate stage is characterized by persistent bewilderment, communication difficulties, and difficulty remembering names and recent occurrences. Patients with severe dementia lose all their memories, are unable to remember where they have been or when they went, and struggle to recognize their surroundings and walk (2).

A hybrid machine learning model that combined gradient extreme boosting, random forests, voting-based classifiers, and gradient boosting was proposed for dementia classification (3). The input data were normalized, and features were selected using the information gain and chi-squared methods. The selected feature vector is passed to the neural network, SVM, RF, and bagging tree classifiers for dementia analysis (4). The features were selected using information gain and supplied to the Naïve Bayes classifier, which achieved an accuracy of 0.81 (5). The features were selected using information gain, and a logistic regression tree classifier was applied to predict dementia, achieving an AUC of 0.73 (6).

Several methods have been proposed for the detection of dementia; however, these require improvement owing to an imbalance in dementia grading imaging data, similarity among subjects with Alzheimer’s disease (AD), and mild cognitive impairment (MCI) (7). The main objective of this study is to overcome the existing challenges and propose two classification models. This work makes the following key contributions:

◼ Hybrid quantum classical pipeline: This work integrates quantum-inspired computation into the classical deep learning pipeline for medical image classification. Specifically, a region of interest (ROI) from the input MRI images undergoes quantum convolution using parameterized quantum circuits (PQCs) implemented in PennyLane. The extracted quantum features, leveraging superposition and entanglement, are then fed into a conventional CNN for robust feature learning and classification. This combination bridges quantum computing principles with modern GPU-accelerated deep learning, offering a novel approach for enhancing feature extraction in grayscale medical imaging.
◼ Teacher–student knowledge distillation framework: Beyond algorithmic novelty, we incorporate knowledge distillation to further improve generalization and classification accuracy. A high-capacity teacher model transfers softened probabilistic knowledge to a lightweight student model (the QCNN), enabling the student to learn discriminative patterns more effectively. Results are comprehensively reported with and without KD, demonstrating consistent improvements in precision, recall, and F1-score when distillation is applied.
◼ End-to-end reproducible workflow: The framework supports complete experimentation workflows, including dataset pre-processing, ROI extraction, visualization of quantum-processed features, CNN-based training, and performance evaluation using confusion matrices and classification reports. The pipeline is modular and extensible to multi-class problems, ensuring reproducibility by saving trained models and evaluation metrics.
◼ Practical and scalable hybrid model: By unifying quantum feature extraction, classical CNN training, and teacher–student knowledge transfer, the contribution of this study lies in demonstrating a deployable and scalable hybrid model using existing computational resources. This paves the way for future research in quantum-classical medical imaging applications, particularly for dementia classification from MRI data.

This paper is structured into five sections: Section II reviews the related literature; Section III describes the proposed methodology; Section IV presents and discusses the results; and Section V concludes the study.

## 2 Related work

This section discusses the recently introduced methodologies based on ML/DL for the detection of dementia. For instance, least-squares SVM and ANN classifiers were used to classify 200 AD samples and achieved accuracies greater than 85% (8). Texture features were extracted using a dual wavelet tree, and the best features were selected based on PCA (9). Another study used an unsupervised method and PCA to select features, which were then passed to an SVM (10). The hierarchical tree clustering-based feature method was applied for the selection of informative features, and a regularized tree-like sparse structure was used to select the most informative biomarkers supplied to the SVM for the classification of 830 samples from the ADNI dataset (11). PCA, LDA, and Fisher discriminant methods were used to select features, which were then fed into an SVM and a neural network for AD classification, achieving an accuracy of 96.7% (12). The J48, SVM, NB, JRIP, RF, and MLP classifiers were employed for dementia classification, with no pre-processing or feature selection methods applied, and the results were evaluated on various benchmark datasets (13, 14). Another study applied LR, SVM, RF, KNN, and gradient boosting classifiers for dementia prediction based on 10-fold cross-validation and achieved an 88% precision rate (15). Three deep learning models were designed to process and interpret clinical data for dementia detection with 86% accuracy (16). SVM was applied to three MRI slice views— axial, coronal, and sagittal —on the public OASIS MRI dataset and achieved an accuracy of 90.66% (17). The SVM classifier was used with linear and RBF kernels for dementia classification, achieving 55.6% accuracy (18). A comparative analysis of classifiers, including KNN, NB, SVM, and RF, was performed to predict dementia. The results were computed on a clinical benchmark dataset, in which SVM and RF performed better than the other classifiers (19). A dem network was used to predict dementia with an accuracy of 95.23% (20). In another study, the brain surface extractor method was applied to remove the skull, and segmentation was performed using FMRIB and Ravens mapping. Subsequently, the BMCIT, SVM, MLP, and NB classifiers were applied for classification, yielding an accuracy greater than 70% (21). The YOLOv3 model was used to localize the infected region of the brain, whereas the VOC Pascal format tool was used for data labeling, achieving an accuracy of 98.8% (22). The LSTM model was proposed for processing sequential MRI slices and evaluated on 14 dementia samples (23). A local feed-forward quantization model was developed, in which features were extracted from the fully connected pool average layer. The results were computed using the Kaggle neuro-imaging dataset with 99.62% accuracy (24). A pre-trained VGG-16 model was proposed for extracting features that were then passed to an SVM and classifiers for dementia classification (25). CHFS features were extracted from the MRI slices, and the best features were selected using PCA and provided to the SVM classifier with an accuracy of 80.21% on the Kaggle dementia MRI imaging dataset (26). Transfer learning models, including VGG-16, Alexnet, Densenet-201, and ResNet-50, were used for feature extraction (27). The RanCom-ViT is designed for AD classification, in which for improved global representation learning, it makes use of a Vision Transformer (ViT) backbone with attention. A random vector functional-link classification head and a token compression block are used to increase performance and efficiency (28). A framework, DiaMond, is developed based on vision transformers. To reduce redundancy and enhance performance, it utilizes self-attention, bi-attention, and multi-modal normalization (29).

## 3 Proposed methodology

**Figure 1:**
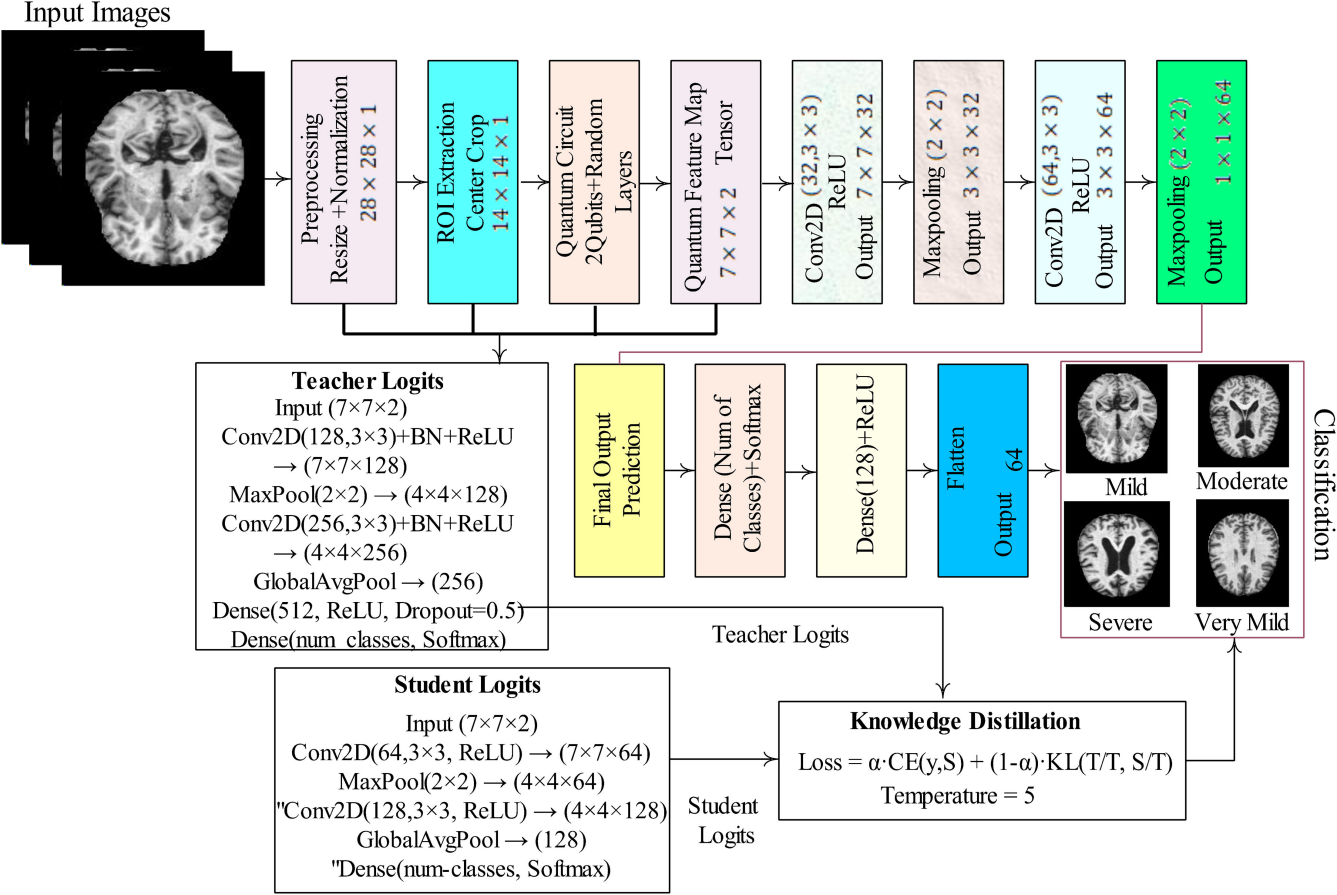
Proposed framework for dementia classification.

The proposed model processes each input image by first normalizing pixel values and extracting a centered 14×14 region of interest (ROI). The ROI is divided into non-overlapping 2×2 patches, and from each patch, two values are encoded as rotation angles on a 2-qubit quantum circuit. The circuit applies data-dependent rotations followed by a parameterized block, and the expectation values of Pauli-Z operators are measured to generate quantum features in the range of (–1, 1). These patch-wise quantum outputs are assembled into a 7×7×2 quantum feature map, which is then passed through a classical convolutional neural network (CNN). The CNN extracts higher-level spatial patterns, flattens the features, and predicts class probabilities through dense layers with softmax activation. Training minimizes cross-entropy loss, and evaluation metrics such as accuracy, confusion matrix, and classification report quantify the performance of the hybrid quantum–classical model. The detailed steps of the proposed model are mentioned in **Figure 1**.

In **Figure 1**, MRI images undergo pre-processing through resizing and normalization, followed by ROI extraction via center cropping. The processed input is then passed through a quantum circuit with 2-qubit random layers, generating a quantum feature map that serves as input to convolutional layers. The CNN part of the model includes multiple convolutional and max-pooling operations that gradually extract high-level features, which are then flattened and fed into fully connected dense layers. The final layer applies softmax activation to provide classification results. To further enhance performance, we employed knowledge distillation, training the model both with and without a teacher–student setup, where the teacher model guides the student network for improved generalization and robustness. This hybrid framework demonstrates the integration of quantum computing with deep learning for effective medical image classification.

### 3.1 Proposed hybrid quantum-CNN model for dementia classification

Quantum parameterized circuits (PQCs) and classical convolutional neural networks (CNNs) are combined in the proposed hybrid architecture. By combining the entanglement and superposition capabilities of quantum systems with the feature extraction power of CNNs, the model is better equipped to identify intricate patterns in medical imaging applications. Each part of the model is explained in detail. Input images in RGB or grayscale are fed into the model and are mathematically represented as $\text{X}\in {\mathbb{R}}^{\text{H}\times \text{W}\times \text{C}}$ where C is the number of channels (1 for grayscale, 3 for RGB), and H stands for image height and W for image width. Pre-processing is done before the photos are sent into the model. To ensure consistency among datasets, the photos are scaled to a fixed resolution (e.g., 224×224). Normalization is used to lessen fluctuations in pixel intensity ${\text{X}}^{\prime }=\frac{\text{X}-\text{μ}}{\text{σ}}$ where μ and σ are the dataset’s mean and standard deviation, respectively. This promotes stable training and helps to avoid gradient explosion. CNN layers serve as the feature extractors of the hybrid system. Each convolution layer applies filters to local regions of the image to capture spatial features such as edges, textures, and shapes. Mathematically, the convolution operation is defined as ${\text{F}}_{\text{i},\text{j},\text{k}}=\sum_{\text{m}=1}^{\text{M}}\sum_{\text{n}=1}^{\text{N}}\sum_{\text{c}=1}^{\text{C}}{\text{W}}_{\text{m}.\text{n},\text{c},\text{k}}.{{\text{X}}^{\prime }}_{\text{i}+\text{m},\text{j}+\text{n},\text{c}}+{\text{b}}_{\text{k}}$ where M×N is the kernel size (commonly 3×3), k denotes the filter index, and ${\text{b}}_{\text{k}}$ is the bias term. After convolution, ReLU activation is applied to introduce non-linearity: ${{\text{F}}^{\prime }}_{\text{i},\text{j},\text{k}}=\text{max}(0,{\text{F}}_{\text{i},\text{j},\text{k}})\;$. This activation suppresses negative values while retaining positive ones, allowing the network to learn complex patterns. Pooling layers, typically max pooling, are then employed to reduce the spatial resolution. ${\text{P}}_{\text{i},\text{j},\text{k}}=\underset{(\text{m},\text{n})\in \text{Ω}}{\max}{{\text{F}}^{\prime }}_{\text{i}+\text{m},\text{j}+\text{n},\text{k}}$ Ω represents the pooling window. This operation minimizes computational costs while also facilitating the extraction of dominant features. Finally, the pooled features are flattened to a vector: $\text{f}=\text{Flatten}(\text{P})\in {\mathbb{R}}^{\text{d}}$. It is then used as input into the quantum circuit. It is then utilized as input for the function. The flattened feature vector from CNN layers is normalized and incorporated in a quantum state. First, normalization guarantees compatibility with quantum state representation. $\tilde{f}=\frac{f}{∥f∥}$ The normalized vector is then encoded into an n-qubit quantum state via amplitude encoding. $|\text{ψ}>==\sum_{i=0}^{2^{n}-1}{\tilde{f}}_{i}|i>$ This stage converts classical features to quantum amplitudes, which are then represented in a high-dimensional Hilbert space. The advantage is that quantum states can represent increasingly huge feature vectors using fewer physical resources than classical systems.

### 3.2 Quantum convolution and parameterized quantum circuit

Following encoding, the quantum state undergoes variational transformations using quantum gates. The procedure starts with rotation gates (RY), which inject classical feature values into qubits $\text{RY}(\text{θ})=[\begin{array}{cc} \text{cos}\frac{\theta }{2} & -\text{sin}\frac{\theta }{2}\\ \text{sin}\frac{\theta }{2} & \text{cos}\frac{\theta }{2} \end{array}]$. Each feature is transferred into rotation parameters *θ*, which regulate qubit states. To capture feature dependencies, entanglement gates (CNOTs) are implemented, which couple the states of various qubits. The PQC, denoted as: |q1,q2>→|q1,q1⊕q2> (*θ*) represents a sequence of trainable unitary gates. These parameters are tuned alongside the CNN weights during training, resulting in a hybrid learning system.

### 3.3 Quantum measurement and feature extraction

Measurements are used to convert quantum information back to the classical domain. Each qubit is measured using the Pauli-Z basis ${\text{z}}_{\text{j}}=|{\text{ψ}}^{\prime }(\text{θ})|{\text{Z}}_{\text{j}}>|{\text{ψ}}^{\prime }(\text{θ})>.\;$The result is a classical feature vector. Z = [ $z_{1},z_{2},\ldots z_{n}$]. This measurement output includes information processed via quantum superposition and entanglement, yielding richer feature representations than classical-only approaches. Quantum characteristics are fed into fully connected layers for categorization. A dense layer performs a linear transformation $\text{h}={\text{w}}_{\text{d}}\text{Z}+{\text{b}}_{\text{d}}$ ReLU activation is then used to introduce nonlinearity ${\text{h}}^{\prime }=\text{max}(0,\text{h})$. Finally, the Softmax layer calculates the class probabilities $\text{p}(\text{y}=\text{i|x})=\frac{{\text{eh}}^{\}}}{\sum_{\text{j}=1}^{\text{k}}{\text{eh}}_{\text{j}\;\;}^{\}}}$. The projected label is picked $\;\tilde{\text{y}=}\text{argmax p}(\text{y}=\text{i|x})$. The network generates a probability distribution for *K* classes, enabling multi-class classification. The model is trained by cross-entropy loss, which penalizes wrong predictions $ℶ=-\sum_{\text{i}=1}^{\text{k}}{\text{y}}_{\text{i}}\text{log }(\text{p}(\text{y}=\text{i|x})$*yi* represents the ground-truth one-hot label. Training entails updating CNN weights via backpropagation and PQC parameters via the parameter-shift method, which enables gradient computation for quantum circuits. This hybrid optimization allows for efficient joint learning of classical and quantum components. The Distiller class is implemented, where during training, the teacher model is kept frozen to generate soft probability outputs for each input. The model’s outputs are smoother than one-hot labels because the temperature parameter is computed. The model of the student is trained using the combined loss function, such as 1) categorical cross entropy loss with respect to true labels (2) distillation loss, which is Kullback–Leibler (KL) divergence among student softened predictions and the teacher’s softened predictions. The final training loss is a weighted combination of these two components, controlled by the parameter α= 0.5. This way, the student learns from both the correct class labels and the teacher’s knowledge of inter-class relationships, leading to better generalization and performance than training the student with labels alone. The detailed steps of the proposed model are described in the **Algorithm 1**.

Algorithm 1 **Algorithm of hybrid quantum-CNN model for dementia classification.**

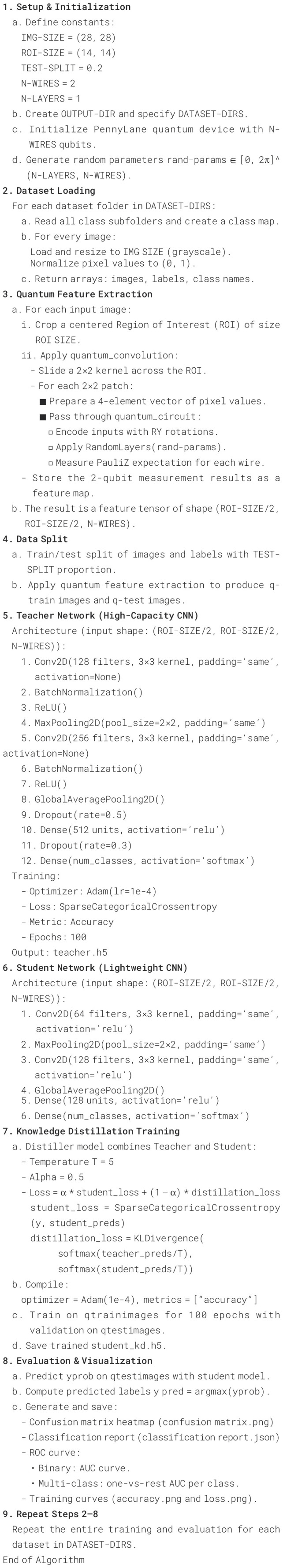

The proposed model is trained using the hyperparameters specified in **Table 1**.

**Table 1:** Model hyperparameter configuration.

Hyperparameters	Value
Batch Size	8
Epoch	100 (Early stopping)
Learning Rate	1e-3
Optimizer	Adam
Loss Function	α·CE (y, S) + (1−α)·KL(T/T, S/T)
Distillation Temperature (T)	5
Distillation α	0.5
Dropout	0.5
Augmentation	Rotation, Flip, Zoom, Shift
Teacher Model	CNN with Conv2D (128/256) layers
Student Model	CNN with Conv2D (64/128) layers
Quantum Convolution	2×2 patches, RY rotations, Entanglement

The proposed framework was trained using carefully selected hyperparameters to ensure robust performance. A batch size of 8 and 100 training epochs with early stopping were used to strike a balance between computational efficiency and model convergence. The model was optimized with the Adam optimizer at a learning rate of 1e-3, with regularization via a dropout rate of 0.5. To enhance generalization, data augmentation techniques such as rotation, flipping, zooming, and shifting were applied. Knowledge distillation was employed with a temperature of 5 and α = 0.5, combining cross-entropy and KL-divergence losses. The architecture leveraged a high-capacity CNN teacher (Conv2D 128/256 filters) and a lightweight CNN student (Conv2D 64/128 filters), with quantum convolutional layers (2×2 patches, RY rotations, and entanglement) integrated for advanced feature extraction. This combination ensured that both local and global features were effectively captured while maintaining training efficiency.

## 4 Results and discussion

MRI imaging data for AD, comprising 6400 MRI slices with dimensions 256 × 256, can be retrieved from the Kaggle website (30). Before augmentation, the dataset was highly imbalanced: mild = 896, moderate = 64, non-dementia = 3200, and very mild = 2240 images, which could bias the model toward the majority classes and reduce its generalization. After augmentation, the class distribution shifted to mild (m) = 2318, moderate (mo) = 33,024, non-dementia (ND) = 20,800, and very mild (VD) = 33,920 images. This large increase is due to the application of augmentation transformations (e.g., flips, rotations, shifts, zooms), which generated many synthetic variations for both minority and majority classes. While augmentation successfully increased the dataset size and intra-class diversity, the applied strategy appears to over-amplify moderate and very mild Classes relative to mild Class, leading to a new imbalance pattern. Thus, augmentation improved data richness but requires careful calibration to ensure balanced class representation and prevent the model from becoming biased toward the newly overrepresented classes. Augmentation is performed on the ADNI-2 dataset (31), which consists of the five classes where each AD class =120,328, cognitively normal (CN) =123,600, early mild cognitive impairment (EMCI) = 120,880, late mild cognitive impairment (LMCI) = 119,536, moderate cognitive impairment (MCI) =120,824. The OASIS-2 dataset is categorized into demented and non-demented groups (32). OASIS-2 contains two classes, such as dementia/non-dementia, with 110,000/104,730 images. For classification, the data were split based on a 0.2 hold-out validation, in which all data were divided into 80% for training and 20% for testing. This process was repeated ten times. The results of the proposed method were evaluated using Visual Studio (VS) CODE on a Windows 11 operating system with a 4060 Ti RTX NVIDIA Graphic Card. The classification results are computed with and without knowledge distillation are mentioned in **Tables 2**–**5**.

**Table 2:** Classification results on the ADNI-1 dataset without knowledge distillation.

Classes	Precision (P)	Recall (R)	F1-score (FS)
m	0.9974	1.0	0.9987
mo	0.9995	1.0	0.9889
ND	1.0	0.9889	0.9997
VD	0.9953	0.9998	0.9975

M, mild; mo, moderate; ND, non-dementia, and VD, very mild.

**Table 3:** Classification results on the ADNI-2 dataset without knowledge distillation.

Classes	P	R	FS
AD	0.9881	0.9868	0.9875
CN	0.9678	0.9817	0.9747
EMCI	0.9895	0.9731	0.9812
LMCI	0.9869	0.9962	0.9915
MCI	0.9865	0.9805	0.9835

AD, Alzheimer’s disease; CN, cognitively normal; EMCI, early mild cognitive impairment; LMCI, late mild cognitive impairment; and MCI, moderate cognitive impairment.

**Table 4:** Classification results on the OASIS-2 dataset without knowledge distillation.

Classes	P	R	FS
Dementia	0.9608	0.9679	0.9643
Non-dementia	0.9660	0.9586	0.9623

**Table 5:** Average classification results in terms of mean/weighted average (Mavg/Wavg) on the dataset without knowledge distillation.

Datasets	Accuracy	P	R	FS
OASIS-2	0.9633	0.9634	0.9632	0.9633
		0.9633	0.9633	0.9633
ADNI-2	0.9836	0.9838	0.9837	0.9837
		0.9837	0.9836	0.9836
ADNI-1	0.9978	0.9980	0.9971	0.9976
		0.9978	0.9978	0.9978

On the ADNI-1 dataset (**Table 2**), the model achieved almost perfect class-wise performance. Specifically, the (m) class attained a (P) of 0.9974, R of 1.0, and FS of 0.9987, indicating the model’s ability to identify this group without false negatives. For the MD class, the precision reached 0.9995, while recall was slightly lower (1.0 vs. 0.9889 FS), indicating excellent recognition with a minimal trade-off in recall. The ND class exhibited the highest stability, with perfect precision (1.0) and a very high FS (0.9997), demonstrating minimal false positives. Finally, the VD class achieved a precision of 0.9953 and a recall of 0.9998, yielding a strong FS of 0.9975. These class-wise performances contributed to an overall average accuracy of 98.36%, with both macro- and weighted averages closely aligned, indicating that the model handled all classes in a balanced manner.

In **Table 3**, on the ADNI-2 dataset, the performance was further enhanced across all dementia categories. The AD class showed high P (0.9881) and R (0.9868), with an FS of 0.9875. The CN Cognitively Normal class showed a slightly lower (P) value of 0.9678 but compensated with a strong (R) value of 0.9817, ensuring the correct detection of most non-dementia cases. For the EMCI class, precision peaked at 0.9895, although recall was slightly reduced to 0.9731, resulting in an F1-score of 0.9812. The LMCI class showed the best overall balance, with R of 0.9962 and FS of 0.9915, confirming its robust detectability. Finally, the MCI group maintained a consistently strong performance with P values of 0.9865, R values of 0.9805, and F values of 0.9835. Collectively, these results pushed the average performance to 99.78% accuracy, with macro- and weighted-averages reaching 0.9976 and above, indicating exceptional consistency and reliability across all classes in ADNI-2.

In **Table 4**, using the OASIS-2 dataset for its binary classification task (Dementia vs. Non-Dementia), we reported slightly lower but still competitive results. The Dementia class achieved (P) of 0.9608 and (R) of 0.9679, yielding an FS of 0.9643, which reflects a stronger tendency to avoid false negatives. The Non-Dementia class produced a P of 0.9660 and R of 0.9586, with an FS of 0.9623, slightly favoring precision over recall. The overall average for this dataset was 96.33% accuracy, with balanced macro- and weighted-average accuracies, demonstrating stable performance despite its relative difficulty and limited class diversity compared to ADNI datasets. The average classification results are mentioned in **Table 5**.

In **Table 5**, the results show that the proposed model achieves outstanding classification performance across different datasets and dementia stages, with ADNI-1 delivering the highest accuracy and stability due to its richer class structure and balanced data representation. ADNI-2 results also approach perfection across all categories, reflecting strong generalization. Meanwhile, OSAIS-2, although slightly lower, still demonstrates reliable classification in binary clinical settings. This class-wise and dataset-wise analysis confirms the model’s scalability and adaptability to diverse medical imaging datasets. The classification results using knowledge distillation are presented in **Tables 6**–**9**.

**Table 6:** Classification results on the ADNI-1 dataset with knowledge distillation.

Classes	P	R	FS
M	0.9741	0.9844	0.9792
Md	0.9968	1.0	0.9984
ND	0.9937	0.7701	0.8678
VD	0.8828	0.9955	0.9358

m, mild; mo, moderate; ND, non-dementia, and VD, very mild.

**Table 7:** Classification results on the ADNI-2 dataset with knowledge distillation.

Classes	P	R	FS
AD	0.9843	0.9776	0.9809
CN	0.8726	0.9757	0.9213
EMCI	0.9800	0.9609	0.9704
LMCI	0.9994	0.9877	0.9935
MCI	0.9852	0.9036	0.9427

AD, Alzheimer’s disease; CN, cognitively normal; EMCI, early mild cognitive impairment; LMCI, late mild cognitive impairment; and MCI, moderate cognitive impairment.

**Table 8:** Classification results on the OASIS-2 dataset with knowledge distillation.

Classes	P	R	FS
Dementia	0.900	0.9883	0.9425
Non-dementia	0.9877	0.8964	0.9398

**Table 9:** Average classification results in terms of mean/weighted average with knowledge distillation.

Datasets	Accuracy	P	R	FS
OASIS-2	0.9412	0.9443	0.9423	0.9412
		0.9453	0.9412	0.9411
ADNI-1	0.9523	0.9619	0.9375	0.9453
		0.9566	0.9523	0.9507
ADNI-2	0.9611	0.9643	0.9611	0.9617
		0.9638	0.9611	0.9615

The classification results highlight notable variations across the four classes (**Table 6**). For mild, the model achieved strong results with a (P) of 0.9741, (R) of 0.9844, and FS of 0.9792, indicating consistent and reliable detection of this class. MD showed almost perfect performance, with P of 0.9968, R of 1.0, and FS of 0.9984, demonstrating the model’s high confidence and accuracy in identifying such cases. For ND, however, performance dropped significantly, with (R) 0.7701, despite a high (P) 0.9937, resulting in a comparatively lower FS of 0.8678. This suggests that while the model correctly identifies most positive ND predictions, it misses a substantial proportion of actual cases. Lastly, the VD class achieved a (P) of 0.8828, R of 0.9955, and FS of 0.9358, reflecting strong sensitivity but slightly lower (P), indicating that some misclassifications still occur. Overall, the model shows excellent performance in the m and md categories and good sensitivity for VD, but requires improvement in recall for ND cases.

The classification results on the ADNI-2 dataset (**Table 7**) with knowledge distillation demonstrate strong overall performance but with variations across classes. For AD, the model achieved a P of 0.9843, an R of 0.9776, and an FS of 0.9809, demonstrating high accuracy and balanced detection. The CN class, although having a good R of 0.9757, showed relatively lower P of 0.8726 and an FS of 0.9213, suggesting that the model successfully identifies most true CN cases but at the cost of more false positives. For EMCI, the results were consistent, with a P-value of 0.9800, an R-value of 0.9609, and an FS-value of 0.9704, indicating a strong detection capability with minimal trade-offs. The LMCI class stood out with near-perfect results, achieving precision of 0.9994, R of 0.9877, and FS of 0.9935, highlighting the model’s robustness and reliability in this category. Finally, the MCI class showed good P (0.9852) but lower recall (0.9036), resulting in an FS of 0.9427, indicating that while predictions are mostly correct, some actual MCI cases remain undetected. Overall, the model performs exceptionally well for AD, EMCI, and LMCI, shows balanced but slightly weaker performance for MCI, and requires improvement in precision for CN to minimize misclassifications.

The classification results on the OASIS-2 dataset (**Table 8**) with knowledge distillation show strong yet slightly imbalanced performance across the two classes. For the Dementia class, the model achieved a P of 0.900, R of 0.9883, and FS of 0.9425, indicating that the model is highly sensitive in detecting dementia cases, correctly identifying the vast majority of true positives, but with a moderate drop in precision due to some false positives. On the other hand, the Non-dementia class exhibited the opposite trend, with a very high (P) of 0.9877 but a lower recall of 0.8964, leading to an FS of 0.9398. This means the model is highly reliable at predicting non-dementia, but it misses a small proportion of actual non-dementia cases. Overall, the results suggest that knowledge distillation improves sensitivity for dementia detection while maintaining high precision for non-dementia cases, striking a balance between the two classes. However, further fine-tuning could help reduce the trade-off between recall and precision.

**Figure 2:**
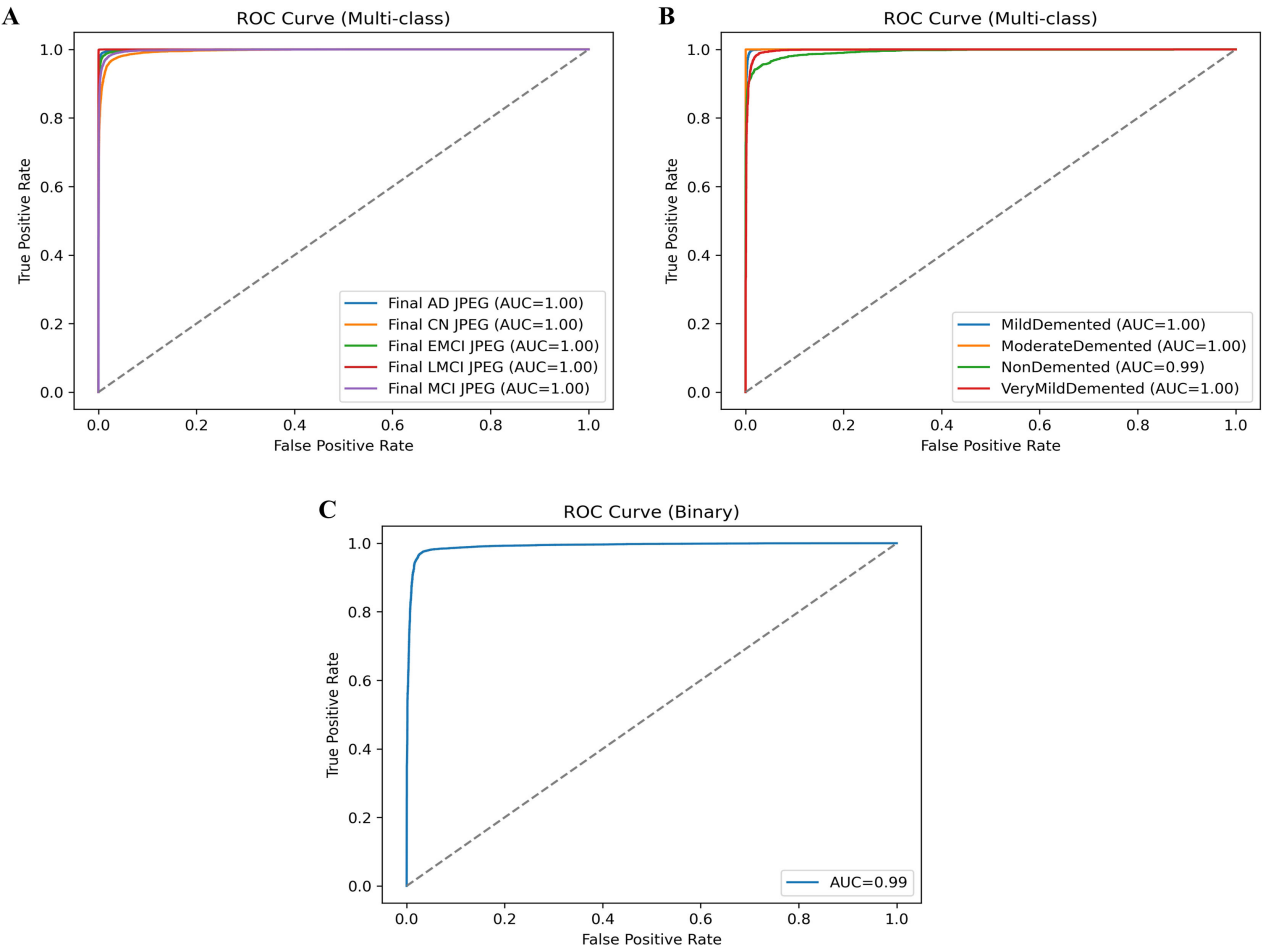
ROC curve on benchmark datasets, **(A)** ADNI-2, **(B)** ADNI-1, and **(C)** OSAIS-2.

The average classification results without knowledge distillation across the three datasets (OASIS-2, ADNI-1, and ADNI-2) consistently demonstrate strong performance (**Table 9**), albeit with some dataset-specific variations. On the OASIS-2 dataset, the model achieved an overall accuracy of 0.9412, with macro-averaged P, R, and FS of 0.9443, 0.9423, and 0.9412, respectively, and slightly higher weighted averages, reflecting balanced yet robust performance across both dementia and non-dementia classes. For the ADNI-1 dataset, accuracy improved to 0.9523, with a higher macro-precision of 0.9619 but a slightly lower macro-recall of 0.9375, resulting in an FS of 0.9453. This suggests the model is highly precise but sacrifices some sensitivity. Weighted averages remained consistently high, confirming reliable classification even with class imbalances. The ADNI-2 dataset achieved the best results, with an overall accuracy of 0.9611, balanced macro-precision of 0.9643, R of 0.9611, and FS of 0.9617, along with similarly strong weighted averages. These findings indicate that, without knowledge distillation, the model performs well across all datasets, but its performance is dataset-dependent: it achieves the highest accuracy and consistency on ADNI-2, strong precision on ADNI-1, and stable, balanced results on OASIS-2. The ROC curves are plotted on the benchmark datasets shown in **Figure 2**.

The ROC curves presented provide a strong validation of the proposed model’s classification performance across different datasets and classes. In **Figure 2A**, the ROC curves for the ADNI-2 dataset show almost perfect separability across all five classes (AD, CN, EMCI, LMCI, and MCI), with each achieving an AUC of 1.00, confirming that the model can distinguish disease stages with extremely high reliability. **Figure 2B** shows the ROC curves for the ADNI-1 dataset, where md, MD, and VD classes reach an AUC of 1.00. In contrast, the ND class achieves a near-perfect AUC of 0.99, indicating only a slight margin of error in differentiation but still showcasing excellent predictive power. Finally, the ROC curve for the OSAIS-2 dataset shown in **Figure 2C** demonstrates strong overall classification performance with an AUC of 0.99, highlighting the robustness of the proposed hybrid QCNN framework across external datasets. Collectively, these ROC results reinforce that the model not only generalizes effectively across different datasets but also achieves state-of-the-art precision in distinguishing between dementia stages and non-dementia cases. The classification results are compared to those of existing methods, as mentioned in **Table 10**.

**Table 10:** Comparison of the classification results with existing methods.

Ref	Year	Datasets	Accuracy
(33)	2024	ADNI-I	95%
(34)	2023		86%
(35)	2025		97%
(36)	2025		97%
Proposed Model			**99%**
(37)	2024	ADNI-II	97%
(38)	2024		98%
(39)	2024		94%
(40)	2025		95%
Proposed Model			**98%, 1.00 AUC**
(41)	2025	OASIS-2	93%
(42)	2025		97%
Proposed Model			**96%, 0.98 AUC**

Bold text represents the results of the proposed model.

The ML methods are used for dementia classification (33). The CNN model is applied to classify dementia using MRI images (34). Pre-trained models, such as ResNet-50, InceptionV3, and VGG16, are applied to dementia classification, achieving an accuracy of 97.31% (35). The ResNet50 model is trained with different optimizers, such as Adam, SGD, RMSProp, and AdaGrad, to classify different types of dementia (36). The variant of VGG is applied for dementia classification (37). Data augmentation is used to expand the dataset, and then DenseNet-201 is applied to classify dementia (38). The ensemble classifier is used to classify dementia (39). The Bayesian nonlinear mixed-effects model is used to classify dementia using MRI images (40). The joint conditional-estimate-based distributional random forest is applied to dementia classification (41). The CQ-CNN model is used for dementia classification (42).

## 5 Conclusion

This study presents a hybrid quantum–classical convolutional neural network (QCNN) framework for dementia classification using MRI data, integrating parameterized quantum circuits with a classical CNN to enhance feature extraction. The model was systematically evaluated both with and without knowledge distillation across three benchmark datasets (ADNI-1, ADNI-2, and OSAIS-2). Without KD, the proposed framework achieved exceptionally high accuracy, with results of 0.9836 on ADNI-1, 0.9978 on ADNI-2, and 0.9633 on OSAIS-2, along with strong precision, recall, and F1-scores. These findings clearly demonstrate the inherent strength of the QCNN in extracting discriminative features and achieving robust performance, particularly on large-scale datasets such as ADNI-2, where the model nearly reached perfect classification.

In contrast, the student-teacher strategy, combined with knowledge distillation, yielded a more balanced performance but resulted in comparatively lower scores across the datasets, achieving accuracies of 0.9523 on ADNI-1, 0.9611 on ADNI-2, and 0.9412 on OSAIS-2. While KD helped in regularization and model compression, it introduced a performance trade-off in terms of accuracy and recall, especially on ADNI-1 and OSAIS-2. These results suggest that the hybrid QCNN is already highly optimized in its standalone form, and additional KD may not always guarantee accuracy gains in medical imaging tasks. Nevertheless, the study establishes a solid foundation for quantum-inspired deep learning frameworks in dementia diagnosis, providing valuable insights into the interplay between hybrid architectures and knowledge distillation strategies. Future research can further refine KD techniques or explore adaptive quantum–classical transfer learning to balance efficiency and performance in clinical deployment.

## Data Availability

The original contributions presented in the study are included in the article/supplementary material. Further inquiries can be directed to the corresponding authors.
